# Ebstein's *Regimen in Gout*

**Published:** 1885-06

**Authors:** 


### Notes on Books.

---

*The Regimen to be adopted in cases of Gout.* By Dr. WILHELM EBSTEIN. Translated by JOHN SCOTT, M.A., M.B. Pp. 68. London: J. & A. CHURCHILL. 1885.

THE aim of the Essay is to deduce rules of practice from pathological facts and theorems. Wherefore, at the beginning the pathological basis is laid. This consists largely in cuttings from the author's treatise on Gout. The pathological introduction is scientific; it is readable and interesting, and asks for assent to no more than can be reasonably supported by scientific evidence. The pathological standpoint may be thus stated: Gout depends upon an increased production of uric acid *in anomalous situations*—a peripheral formation of uric acid in muscles and the medulla of bones—with secondary affections of joints, kidneys, and organs of circulation. The increased formation of uric acid depends on *predisposition*, probably always inherited. This view is taken in opposition to the theory that gout depends on retention of uric acid from defective elimination through the kidneys.

Wherefore, the author gains the first indication for treatment; viz., to procure a reduction of uric acid formation in general, and thus to prevent the particular production in anomalous situations.

The rest of the Essay is devoted to a consideration of the regimen best suited to the gouty, especially in the prophylaxis of gout. Food—its nature and quantity; drink—alcohol, water, &c.; baths; clothing; exercise—each of these topics is carefully and temperately discussed. In speaking of food, the author combats the generally received notion that fat is bad for the gouty; he advocates the use of fat, for the reason (amongst other reasons) that it does not provoke dyspepsia, but, on the contrary, often relieves dyspepsia when substituted for part of the starch in a diet which has been too starchy. On the whole, the regimen advocated is an easy and comfortable one; and its adoption would tend, in our opinion, to make many unfortunates to rejoice who have hitherto led miserable lives in the endeavour, unsuccessfully, to prevent in their bodies the formation of any uric acid at all. The author goes well to the root of the matter of prophylaxis, for he enjoins on gouty parents the duty of attending at an early period to the dietary and training of their children.

The value of the book is enhanced, and it is made more readable, by placing at the end of the Essay, as "Additions and Illustrations," discussions on scientific questions which arise in the course of the text.

The Translator has done his work admirably.

